# *RAD51* and* XRCC3* Polymorphisms Are Associated with Increased Risk of Prostate Cancer

**DOI:** 10.1155/2019/2976373

**Published:** 2019-05-02

**Authors:** Maria Nowacka-Zawisza, Agata Raszkiewicz, Tomasz Kwasiborski, Ewa Forma, Magdalena Bryś, Waldemar Różański, Wanda M. Krajewska

**Affiliations:** ^1^Department of Cytobiochemistry, Faculty of Biology and Environmental Protection, University of Lodz, Lodz, Poland; ^2^Department of Urology 2, Faculty of Biomedical Sciences and Postgraduate Training, Medical University of Lodz, Lodz, Poland

## Abstract

Genetic polymorphisms in DNA repair genes may affect DNA repair efficiency and may contribute to the risk of developing cancer. The aim of our study was to investigate single nucleotide polymorphisms (SNPs) in* RAD51* (rs2619679, rs2928140, and rs5030789) and* XRCC3 *(rs1799796) involved in DNA double-strand break repair and their relationship to prostate cancer. The study group included 99 men diagnosed with prostate cancer and 205 cancer-free controls. SNP genotyping was performed using the PCR-RFLP method. A significant association was detected between* RAD51* rs5030789 polymorphism and* XRCC3* rs1799796 polymorphism and an increased risk of prostate cancer. Our results indicate that* RAD51* and* XRCC3* polymorphism may contribute to prostate cancer.

## 1. Introduction

Prostate cancer is the second most commonly occurring cancer and the fifth leading cause of cancer death in men with an estimated 1.3 million new cases and 359.000 associated deaths worldwide in 2018. It is the most frequently diagnosed cancer among men in over one-half of the countries of the world [1, 2]. Prostate cancer is characterized by the highest dynamic of increase in the last decade, and in 2016, for the first time, it became the most common cancer among men in Poland [3]. This cancer is very rarely manifested before the age of 50, and more than half of patients at the time of diagnosis are at least 70 years old. Age-adjusted incidence rates of prostate cancer increased dramatically and this is largely because of the increased availability of screening for specific prostate antigen (PSA) in men without symptoms of the desease. PSA screening offers a potential benefit of reducing the chance of death from prostate cancer. However, the value of PSA screening is moderate. An increase in PSA over 4 ng/ml suggests cancer, but nearly 25% of men with elevated levels of PSA do not have cancer, and nearly 20% of patients with prostate cancer have normal serum PSA. Elevated PSA levels may be also associated with benign conditions such as inflammation and benign prostatic hypertrophy and procedures such as bladder catheterization, transrectal ultrasound, gland biopsy, cystoscopy, and transurethral endoscopy. The search for markers other than PSA, allowing for early diagnosis and prognosis of prostate cancer, seems to be justified [3, 4]. The factors associated with an increased risk of prostate cancer include family burden, race, ethnicity, obesity, high fat diet, smoking, and exposure to androgens [2]. Germline and somatic mutations appeared to be well-established risk factors for primary and metastaic prostate cancer. In addition, genome-wide association studies (GWAS) have identified approximately 170 SNPs associated with the development of prostate cancer. Pathogenic variants of high and moderate penetrance genes, such as* BRCA1* and* BRCA2*, mismatch repair genes, and* HOXB13* confer modest to high lifetime risk of prostate cancer. Some, such as* BRCA2*, have emerging clinical relevance in the treatment and screening for prostate cancer [5–8].

The process of tumorigenesis occurs in the absence of efficient DNA repair systems and this may, among others, result from genetic variations in the genes involved in them. The most deleterious form of DNA damage is the double-strand break (DSB). In order to maintain genomic stability, double-strand breaks must be repaired by homologous recombination (HR) or nonhomologous end joining (NHEJ). Germline and somatic mutations in genes that promote homology-directed repair, especially* BRCA1* and* BRCA2*, are frequently observed in several cancers, in particular, breast and ovary, but also prostate and other cancers. The critical biochemical function of BRCA2 in homology-directed repair is to promote RAD51 filament assembly onto ssDNA that arises from end resection. BRCA2 directly interacts with RAD51 at multiple sites to facilitate RAD51 filament assembly. BRCA2 is shown to regulate both the intracellular localization and DNA-binding ability of RAD51. Loss of these controls may be a key event leading to genomic instability and tumorigenesis [9, 10]. The human* RAD51*, located on chromosome 15q15.1, plays a crucial role in DNA double-strand break repair [11]. The protein encoded by this gene is a member of RAD51 protein family. RAD51 family members are highly similar to bacterial RecA and* Saccharomyces cerevisiae* Rad51 and are known to be involved in the homologous recombination and repair of DNA. RAD51 binds to single- and double-stranded DNA and exhibits DNA-dependent ATPase activity. RAD51 catalyzes the recognition of homology and strand exchange between homologous DNA partners to form a joint molecule between a processed DNA break and the repair template. RAD51 binds to single-stranded DNA in an ATP-dependent manner to form nucleoprotein filaments which are essential for the homology search and strand exchange. RAD51 plays a role in regulating mitochondrial DNA copy number under conditions of oxidative stress in the presence of RAD51C and XRCC3 and is also involved in interstrand cross-link repair. At the site of DNA damage nuclear foci containing BRCA1, BRCA2, and RAD51, together with other proteins engaged in homologous recombination, are forming. The protein that binds to RAD51 is XRCC3. This combination facilitates formation of the nucleoprotein filament that represents primary vector for both homologous and heterologous recombination [12–16].

As we have previously shown the rs1801320 polymorphism in* RAD51* may contribute to prostate cancer susceptibility in Poland [17]. The purpose of the presented work was to investigate further selected single nucleotide polymorphisms (SNPs), i.e., rs2619679, rs2928140, and rs5030789 in* RAD51* and rs1799796 in* RAD51 *paralog* XRCC3* and their relationship to prostate cancer.

## 2. Material and Methods

### 2.1. Patients

The study group included 99 men with prostate adenocarcinoma and 205 sex- and age-matched cancer-free subjects with low (<4 ng/ml) levels of PSA as a control group. Peripheral blood samples from the patients with prostate adenocarcinoma were obtained from the Department of Urology 2, Medical University of Lodz, Poland. Peripheral blood samples from the control group were obtained from the Urological Department of the Provincial M. Sklodowska-Curie Hospital in Zgierz, Poland.  Table 1 presents clinicopathological characteristics of patients and the control group.

### 2.2. DNA Isolation

DNA from peripheral blood was isolated by phenol extraction [18] or using AxyPrep Blood Genomic DNA Miniprep Kit (Axygen Biosciences) and stored in -70°C. DNA preparations were subjected to spectrophotometric analysis (Biophotometer Eppendorf AG, Germany) by measuring absorbance at 260 nm and 280 nm to determine the quantity and quality of the isolated nucleic acid. The A260/A280 ratio was in the range 1.8-2.1.

### 2.3. Genotyping

Single nucleotide polymorphism (SNP) was determined by PCR-RFLP (polymerase chain reaction-restriction fragment length polymorphism). Tested SNPs are shown in the Table 2.

The primers for studied SNPs were as follows: (F) 5′-CCGTGCAGGCCTTATATGAT-3′ and (R) 5′-AGATAAACCTGGCCAACGTG-3′ for rs2619679; (F) 5′-GCTTCTGGCTATTTTCAAGT-3′ and (R) 5′-TGAGGCAGGTAAATGGCTTC-3′ for rs2928140; (F) 5′-CCGTGCAGGCCTTATATGAT-3′ and (R) 5′-AGATAAACCTGGCCAACGTG-3′ for rs5030789; (F) 5′-CCGCATCCTGGCTAAAAATA-3′ and (R) 5′-CAGAGTATGGGCACTGTGAGC-3′ for rs1799796. The primers were synthesized at Sigma-Aldrich®. The polymerase chain reaction (PCR) was performed in an Applied Biosystems® 2720 thermocycler in total volume of 10 *μ*l. The reaction mixture contained 10 ng of genomic DNA; 0.2 *μ*moles of primers (F) and (R); 3 HOT FIREPol® units of DNA polymerase (5 U/ml); 1 mM GeneAmp dNTPmix (10 mM); 2.5 mM magnesium chloride (25 mM); and 1 x Solis BioDyne buffer B1 (10x concentrated). The components of the PCR reaction mixture were from Solis BioDyne (Estonia) and Applied Biosystem (USA).

The temperature-time profile of PCR was as follows: Pre-PCR: 95°C for 12 min; PCR (30 cycles): 95°C for 0.5 min, 63°C (rs2928140) or 64°C (for rs2619679 and rs1799796) or 65°C (rs5030789) for 0.5 min, 72°C for 1 min; Post-PCR at 72°C for 5min.

**Figure 1:**
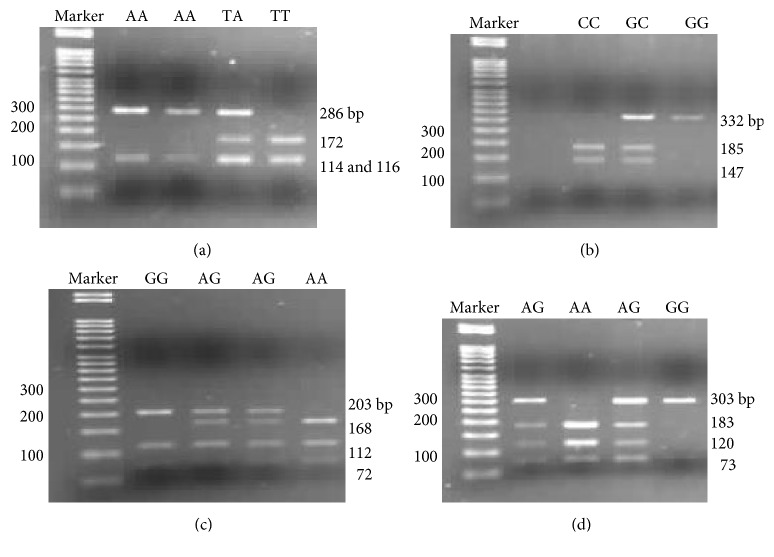
PCR-RFLP genotyping of (a)* RAD51* rs2619679 polymorphism; (b)* RAD51* rs2928140 polymorphism; (c)* RAD51* rs5030789 polymorphism; (d)* XRCC3* rs1799796 polymorphism.

The amplification products were digested with restriction enzymes:* Hinf*I (rs2619679),* Ear*I (rs2928140),* Nla*III (rs5030789), or* Alu*I (rs1799796) at 37°C for 16 hours. Enzyme inactivation lasted 20 minutes at 65°C for* Ear*I and at 80°C for* Hinf*I,* Nla*III, and* Alu*I. The enzymes came from New England BioLabs Inc. DNA fragments were separated in a 3% agarose gel with ethidium bromide for UV visualization. Electrophoresis was performed in 1x TBE buffer (10x TBE: 89 mM Tris, 89 mM boric acid, 2 M EDTA pH 8.0) and 100V. Examples of the obtained restriction patterns are shown in Figure 1.

### 2.4. Statistical Analysis

The compatibility of the genotype distribution with the Hardy-Weinberg law in the control group and in study group was checked by the *χ*^2^ test. Significance of differences between the distribution of genotypes/alleles in the control and study group was assessed by the *χ*^2^ test. The risk of comorbidity of genotypes/alleles with the disease was assessed based on odds ratio (OR) together with a 95% confidence interval. All results were considered statistically significant at* p* values <0.05. Statistical calculations were made using spreadsheets available on the websites: quantpsy.org/chisq/chisq.htm and vassarstats.net/odds2x2.html.

## 3. Results

Table 3 presents results of studied polymorphisms in* RAD51* and* XRCC3* using the PCR-RFLP method. The distribution of genotypes and alleles in the control group and in patients with prostate cancer was consistent with Hardy-Weinberg law (*p*>0.05). Statistically significant differences were found in the distribution of genotypes and alleles for rs5030789 and rs1799796 polymorphism in* RAD51* and* XRCC3*, respectively, between control group and prostate cancer patients.

The odds ratio (OR) analysis showed that rs5030789 polymorphism in* RAD51* and rs1799796 polymorphism in* XRCC3* are associated with susceptibility to prostate cancer (Table 4). The presence of the GG genotype in both polymorphic sites of* RAD51* and* XRCC3* increases the risk of prostate cancer (OR = 2.782,* p* = 0.038 for rs5030789; OR = 1.986,* p* = 0.041 for rs1799796). Also, the presence of the G allele increases the risk of developing prostate cancer in both above polymorphisms (OR = 1.571 for rs5030789 and OR = 1.441 for rs1799796,* p*<0.05).

Because the polymorphism rs5030789 in* RAD51* and polymorphism rs1799796 in* XRCC3* increase the risk of prostate cancer, the correlation of these polymorphisms with age and clinicopathological characteristcs of prostate cancer patients was examined (Table 5). It was revealed that there is a relationship between rs1799796 polymorphism in* XRCC3* and the age of patients over 71 years (OR = 1.916,* p* = 0.033) and Gleason score of cancer equal to or higher than 7 (OR = 2.373,* p* = 0.012). No association was found with the level of PSAT, nor with rs5030789 in* RAD51* nor rs1799796 in* XRCC3*.

## 4. Discussion

Prostate specific antigen (PSA) is a blood-based biomarker used for the detection and surveillance of prostate cancer. However, PSA levels can also be affected by benign prostatic hyperplasia (BPH), local inflammation or infection, prostate volume, age, and genetic factors. In this regard, PSA seems to be an organ but not cancer specific biomarker [19]. Seeking the molecular mechanisms underlying prostate cancer, many mutations and polymorphisms of a single nucleotide have been identified, especially in DNA repair genes, which increase the risk of developing prostate cancer. Polymorphic genes of DNA repair are in great part included in low penetrance genes, which means that single gene product most often slightly affects the disease occurrence risk, but accumulation of changed alleles can have essential significance for its development. RAD51, which is a critical protein involved in the homologous recombination repair pathway, interacts with XRCC2, XRCC3, and other proteins to form a complex that is crucial for repairing the double-strand breaks and maintaining chromosome stability [12, 16, 20].

To our knowledge, genetic abnormalities in RAD51 paralogs, i.e., RAD51C and RAD51D, have been identified in prostate cancer, but not in RAD51 [5–10]. Our study has shown the importance of* RAD51 *and its paralog* XRCC3* polymorphism in prostate cancer. Single nucleotide polymorphism within these genes may affect DNA double-strand break repair capacity, hence the increased susceptibility to neoplastic transformation. There is growing body of evidence which suggests that polymorphic variants of these genes have impact on developing different cancers. A meta-analysis conducted by Zeng et al. [11] suggests that* RAD51 * rs1801320 (135G/C) polymorphism is a risk factor for three common gynecological tumors, i.e., breast, endometrial, and ovarian cancers, and especially for endometrial cancer. Al-Zoubi et al. [21] in their studies demonstrated that the homozygous variant T172T (rs1803121) is significantly associated with breast cancer risk (OR 3.717, 95% CI 2.283-6.052,* p* < 0.0001), while the heterozygous variant G135C (rs1801320) has no significant relationship with the risk of sporadic breast cancer (OR 1.598, 95% CI 0.5638-4.528,* p* > 0.05). However, both variants homozygous T172T and heterozygous G135C together showed a significant association with sporadic breast cancer susceptibility. Michalska et al. [22] found that the polymorphism of* RAD51* may be positively associated with the incidence of triple-negative breast carcinoma while Sekhar et al. [23] indicated that* RAD51* 135G > C substitution in the homozygous form (CC) increases the risk of breast cancer in an ethnic-specific manner. Söderlund et al. [24] suggest that* RAD51* 135G>C polymorphism predicts cyclophosphamide/methotrexate/5-fluorouracil chemotherapy effect in early breast cancer.

Polymorphism of the* RAD51* also seems to play a role in other types of cancer. In our previous study we found a significant relationship between* RAD51* polymorphism rsl801320 and an increased risk of prostate cancer [17]. It has been shown that subjects carrying* RAD51* rs1801320 GC genotype also have an increased risk of glioblastoma (GC vs GG, *χ*(2) = 10.75; OR 3.0087;* p* = 0.0010). In addition,* RAD51* rs1801320 C allele increased the risk of developing glioblastoma also in combination with the* XRCC1* rs25487 G allele and* XRCC3* rs861539 C allele (*χ*(2) = 6.558;* p* = 0.0053) [25]. Trang et al. [26] showed that the combination of* Helicobacter pylori* infection and* RAD51* G135C genotype of the host leads to an increased score for intestinal metaplasia. This suggests that* RAD51* G135C may be an important predictor for gastric cancer of* Helicobacter pylori*-infected patients. Mucha et al. [27] study revealed a statistically significant association also between rs5030789 polymorphism in* RAD51* and the risk of colorectal cancer. In turn in the case of rs2619679 polymorphism in* RAD51*, it was shown that it does not correlate with the risk of head and neck cancer [28].

Avadanei et al. [29] findings suggest that* XRCC3* polymorphism in hepatocellular carcinoma may affect the aggressiveness of the tumor expressed by tumor grade. Statistically significant differences were shown for rs1799796 A>G and tumor grade, between wild type (AA) and heterozygote (AG) genotypes, and wild type (AA) and heterozygote and homozygote (AG and GG) genotypes. The logistic regression analysis found an OR of rs1799796 polymorphism occurrence in hepatocellular carcinoma related to tumor grade. In the case of rs861539 C>T polymorphism, statistical analysis showed better survival only for the homozygote (TT) compared to the heterozygote (CT) genotype, and in the case of rs1799796 A>G polymorphism, a longer survival for wild type (AA) compared to heterozygote (AG) and to heterozygote and homozygote (AG and GG) genotypes, respectively. The results presented by Ali et al. [30] suggest that the polymorphism rs1799794 in* XRCC3* is strongly associated with the development of breast cancer in Saudi women while genotype and allele frequencies of rs861539 C>T and rs1799796 A>G did not show a significant difference. However, the frequency of rs1799796 differed significantly in patients depending on the age of the diagnosis, tumor grade, and ER and HER2 status. The wild type A allele occurred more frequently in the ER- and HER2- group. It was also found that the presence of the polymorphism rs1799796 in* XRCC3* may reduce the risk of oral premalignant lesions [31]. On the other hand, Mandal et al. [32] showed no significant association between rs1799796 and rs861539 polymorphism in* XRCC3* and the risk of prostate cancer. In the case of studies conducted by Mittal et al. [33], no direct relationship was found between the occurrence of rs1799796 polymorphism in* XRCC3* and the incidence of bladder cancer. In addition, the studied polymorphism seems to be not related to the incidence of nasopharyngeal cancer as well as head and neck cancer [27, 34]. However, a meta-analysis of 5302 cases of ovarian cancer compared to 8075 control cases revealed statistically significant correlation of rs1799794 and rs1799796 polymorphism in* XRCC3* and an increased risk of developing ovarian cancer in Caucasians, Asian, and African population [35]. It is also worth pointing out that Vral et al. [36] have demonstrated the combined effect of polymorphisms in* RAD51* and* XRCC3* on breast cancer risk.

## 5. Conclusion

Our study showed that rs5030789 polymorphism in* RAD51* and rs1799796 in* XRCC3* are associated with the occurrence of prostate cancer in Polish men. We have demonstrated correlation between the rs1799796 polymorphism in* XRCC3* and the age of patients over 71 years and Gleason score of tumor higher than 7. Our findings indicate the importance of* RAD51* and* XRCC3 *polymorphisms in the development of prostate cancer. Based on the results presented, we suggest considering genetic testing for* RAD51* and* XRCC3* to identify those men who have DNA repair deficiency and who have not responded to standard treatment.

## Figures and Tables

**Table 1:** Clinicopathological characteristics of studied material.

	Parameter
Control group (n=205)	

*Age*	
Range	43 - 84
Mean ± SD	63.33 ± 9.28
Median	64

*PSAT (ng/ml)*	
Range	0.004 – 3.94
Mean ± SD	1.09 ± 0.88
Median	0.95

Patients with prostate cancer (n=99)	

*Age*	
Range	49 - 85
Mean ± SD	70.38 ± 8.63
Median	71

*PSAT (ng/ml)*	
Range	4.01 – 1489.00
Mean ± SD	59.17 ± 184.59
Median	9.22

*Free/total PSA (F/T PSA)*	
Range	0.04-0.79
Mean ± SD	0.19±0.15
Median	0.16
< 0.16	48
≥ 0.16	51

*PSA Density (PSAD, ng/ml)*	
Range	0.07-56.4
Mean ± SD	2.57±8.44
Median	0.28
< 0.28	49
≥ 0.28	50

*Prostate volume (ml)*	
Range	20.7-191
Mean ± SD	59.5±39.0
Median	48.2
< 48	46
≥ 48	53

*Gleason score*	
< 7	28
≥ 7	71

*Cancer stage*	
T1-T2	58
T3-T4	41

**Table 2:** Polymorphic sites in *RAD51* and *XRCC3* (according to NCBI).

Gene	SNP	Other names	Chromosome	SNP position
*RAD51*	rs2619679	g.3879T>A	15: 40694039	Promoter
		c.-1285T>A		
	rs2928140	g.7995G>C,	15: 40698155	Intron 1
		c.-2-602G>C		
	rs5030789	g.3997A>G,	15: 40694157	Promoter
		c.-1167A>G		

*XRCC3*	rs1799796	g.20897A>G	14: 103699590	Intron 7
		c.562A>G		

**Table 3:** Distribution of genotypes and allele frequency of studied SNPs in *RAD51* and *XRCC3* in prostate cancer patients and control group.

Gene	rs	Genotype/allele	Control group (n=205)	Prostate cancer patients (n=99)
*RAD51*	rs2619679	TT	48	30
		TA	101	51
		AA	56	18
		*χ* ^2^ = 3.59, *p* = 0.17		
		T	197	111
		A	213	87
		*χ* ^2^ = 3.43, *p* = 0.06		
	rs2928140	GG	95	43
		GC	63	36
		CC	47	20
		*χ* ^2^ = 1.00, *p* = 0.61		
		G	253	122
		C	157	76
		*χ* ^2^ = 0, *p* = 1.00		
	rs5030789	AA	29	7
		AG	106	45
		GG	70	47
		*χ* ^2^ = 6.43, *p* = *0.04*		
		A	164	59
		G	246	139
		*χ* ^2^ = 5.98, *p* = *0.01*		

*XRCC3*	rs1799796	AA	77	28
		AG	92	45
		GG	36	26
		*χ* ^2^ = 4.15, *p* = 0.13		
		A	246	101
		G	164	97
		*χ* ^2^ = 4.40, *p* = *0.04*		

**Table 4:** Prostate cancer risk and *RAD51* and *XRCC3* polymorphism.

Gene	rs	Genotype/allele	Control group (n=205)	Prostate cancer patients (n=99)	OR (95% Cl)	*p* value
*RAD51*	rs2619679	TT	48	30	1 (Ref.)	
		TA	101	51	0.808 (0.459-1.424)	0.554
		AA	56	18	0.514 (0.255-1.036)	0.089
		T	197	111	1 (Ref.)	
		A	213	87	0.725 (0.515-1.020)	0.077
	rs2928140	GG	95	43	1 (Ref.)	
		GC	63	36	1.262 (0.732-2.178)	0.484
		CC	47	20	0.940 (0.498-1.775)	0.841
		G	253	122	1 (Ref.)	
		C	157	76	1.004 (0.708-1.423)	0.526
	rs5030789	AA	29	7	1 (Ref.)	
		AG	106	45	1.759 (0.718-4.309)	0.299
		GG	70	47	*2.782 (1.126-6.872)*	*0.038*
		A	164	59	1 (Ref.)	
		G	246	139	*1.571 (1.093-2.228)*	*0.018*

*XRCC3*	rs1799796	AA	77	28	1 (Ref.)	
		AG	92	45	1.345 (0.768-2.356)	0.371
		GG	36	26	*1.986 (1.022-3.860)*	*0.041*
		A	246	101	1 (Ref.)	
		G	164	97	*1.441 (1.024-2.027)*	*0.044*

**Table 5:** Relationship between G allele for rs5030789 in *RAD51* and rs1799796 in *XRCC3* and clinicopathological characteristics of prostate cancer patients.

Clinicopathological parameter	rs5030789		rs1799796	
	A	G	A	G
Age				
≤ 71	35	67	60	42
> 71	24	72	41	55
	OR = 1.567 (0.846-2.902)		*OR = 1.916 (1.089-3.371)*	
	*p* = 0.202		*p = 0.033*	

PSAT (ng/ml)				
< 4-10	34	68	53	49
> 10	25	71	48	48
	OR = 1.420 (0.768-2.624)		OR = 1.082 (0.619-1.889)	
	*p* = 0.335		*p* = 0.887	

Free/total PSA (F/T PSA)				
< 0.16	25	71	44	52
≥ 0.16	34	68	57	45
	OR = 0.704 (0.381-1.301)		OR = 0.668 (0.381-1.170)	
	*p* = 0.335		*p* = 0.203	

PSA Density (PSAD, ng/ml)				
< 0.28	26	72	49	49
≥ 0.28	33	67	52	48
	OR = 0.733 (0.397-1.352)		OR = 0.923 (0.529-1.612)	
	*p* = 0.399		*p* = 0.888	

Prostate volume (ml)				
< 48	31	61	52	40
≥ 48	28	78	49	57
	OR = 1.416 (0.768-2.608)		OR = 1.512 (0.862-2.652)	
	*p* = 0.337		*p* = 0.192	

Gleason score				
< 7	19	37	37	19
≥ 7	40	102	64	78
	OR = 1.309 (0.675-2.541)		*OR = 2.373 (1.246-4.521)*	
	*p* = 0.532		*p = 0.012*	

Cancer stage				
T1-T2	35	81	58	58
T3-T4	24	58	43	39
	OR = 1.224 (0.664-2.256)		OR = 0.907 (0.515-1.597)	
	*p* = 0.624		*p* = 0.841	

## Data Availability

The data used to support the findings of the study are included within article.
